# A review on post-COVID-19 impacts and opportunities of agri-food supply chain in Malaysia

**DOI:** 10.7717/peerj.15228

**Published:** 2023-05-01

**Authors:** Say Peng Tan, Lee Chuen Ng, Novel Lyndon, Zaki Aman, Parthiban Kannan, Khairuman Hashim, Han Meng Teo, Muhamad Syazlie Che Ibrahim

**Affiliations:** 1 Malaysian Palm Oil Board (MPOB), Bandar Baru Bangi, Selangor, Malaysia; 2 Faculty of Social Sciences and Humanities, Universiti Kebangsaan Malaysia, Bangi, Selangor, Malaysia; 3 Faculty of Fisheries and Food Science (FFFS), Research Interest Group of Resource Sustainability (Bio-interaction and Crop Health), Laboratory of Pest, Disease and Microbial Biotechnology (LAPDiM), Universiti Malaysia Terengganu, Kuala Nerus, Terengganu, Malaysia

**Keywords:** Post COVID-19, Agri-food, Pandemic, Food supply chain, Livelihood

## Abstract

**Background:**

Malaysia is strongly supported by the agriculture sector as the backbone to drive the economy. However, COVID-19 has significantly affected agriculture across the production, supply, and marketing chains. It also disturbs the balance of food supply and demand in Malaysia. COVID-19 was an unexpected pandemic that resulted in shock and panic and caused a huge global impact. However, the impacts of this pandemic on the agriculture sector in Malaysia, particularly in the production and supply chains, are still unclear and scarce. This review offers insights into the challenges, particularly in sustaining agri-food production and supply chains. It also highlights the opportunity and relevant measures towards sustainability in agriculture to avoid agri-food disasters in the future.

**Methods:**

This study was carried out through a desk review of the secondary source of information covering the impact of COVID-19 in Malaysia particularly in the agri-food aspect, and a wide range of strategies and initiatives as the effective measures to overcome the crisis of this pandemic. Online desk research of the government published data and customer desk research were utilized to complete this study. Search engines such as Google Scholar and the statistical data from the official websites including the Department of Statistics Malaysia (DOSM) and the Food and Fertilizer Technology Center for the Asian and Pacific Region (FFTC-AP), were utilized. Keywords such as impact of COVID-19, pandemic, and agri-food supply chain were used to conduct the searches. The articles identified to be related to the study’s objective were then downloaded and included in the study. Descriptive methods were used as the primary analysis technique following the descriptive analysis and visual data analysis in performing the sources obtained.

**Results:**

This devastating impact damages the lives by causing 4.3 million confirmed infections and more than 290,000 deaths. This disease presents an unprecedented challenge to the public health. The lockdown restriction under the movement control order (MCO), for more than of the world’s population in the year 2020 to control the virus from spreading, has disrupted most of the economic sectors. The agriculture industry was seen as one of the essential industries and allowed to operate under strict standard operating procedures (SOP). Working under strict regulations came with a huge price paid for almost all industries.

**Conclusion:**

This pandemic has affected the national agri-food availability and accessibility in Malaysia. This outbreak created a reflection of opportunity for sharing a more flexible approaches in handling emergencies on agricultural food production and supply chains. Therefore, the government should be ready with the roadmap and enforce the measures to control the pandemic without disrupting the agri-food supply chain in the near future.

## Introduction

The resilience in crisis of agriculture industry during the pandemic of COVID-19 is crucial to minimize the severe consequences for other industries to overcome the short, medium and long-term impacts. The impact of COVID-19 forced the decreasing in the planted area of some important commodities and cash crops such as palm oil, cocoa, paddy, coconut and mustard between 2019–2021 (Table 1). However, the impacts of this pandemic on the agriculture sector in Malaysia, particularly in the production and supply chains are still unclear and scarce. This study comprehends the impacts of COVID-19 on the agriculture sector in Malaysia especially in the agri-food production and supply chains. The study also summarizes the effective alternate strategies that have been implemented by the government of Malaysia and the transformation by the agri-food industry to sustain the agri-food supply chain. This review also highlights the opportunity and relevant measures towards sustainability in agricultural production system to avoid agri-food crises in the future for the next coming pandemic.

**Table 1:** Planted area for main crops in Malaysia, 2018–2021. Impact of COVID-19 pandemic resulted in decreasing of crop planted area between year 2020–2021.

Crops	2018	2019	2020	2021
Oil palm	5,849.3	5,900.2	5,865.3	5,737.7
Cocoa	15.6	5.9	5.7	6.0
Paddy	700.0	672.1	644.9	647.9
Coconut	83.4	86.5	84.9	82.6
Mustard	9.4	11.2	10.9	11.4

**Note:** (′000 hectares)

Source: Department of Statistics Malaysia (Department of Statistics Malaysia (DOSM), 2021a).

COVID-19 caused by the SARS-CoV-2 virus that disturbs the respiratory system through difficulty in breathing, fever and cough (Christian, 2020) was firstly reported in December 2019, as a new acute respiratory infections in Wuhan City, Hubei Province (Elengoe, 2020). COVID-19 was found to cause a high mortality rate among adults with cancer (Rouger-Gaudichon et al., 2020) and the elderly (Henkens et al., 2021). Death was inevitable for some patients who were infected with low immune systems (Begum et al., 2021). Globally, the COVID-19 pandemic had caused more than 4.3 million confirmed infections and more than 290,000 deaths (Nicola et al., 2020). As the number of infected and dead increased, the fear of economic crisis and recession also increased. This virus was reported to be transmitted through direct contact to the respiratory droplets of the infected COVID-19 patient or indirect contact with virus-contaminated surfaces (Wu, Chen & Chan, 2020). Airborne transmission of this virus is possible with the presence of microbes within droplet nuclei that remain in the air (World Health Organization (WHO), 2020). The first transmission of COVID-19 through food was first associated to the seafood market in Hunan (one of the cities in Wuhan) China (Li et al., 2020). On 14 September 2022, the cases of COVID-19 had reached over 609 million and over 6.5 million corresponding deaths were reported (ArcGIS, 2022).

The rapid spread of COVID-19, followed by a total lockdown, was a huge surprise for the global economy, including Malaysia. The pandemic of COVID-19 had tremendously affected the society in the aspects of environmental and economical (Kluge et al., 2020). The enforcement of lockdown and mobility restriction had worsen the pandemic impacts in the agricultural businesses and food industries (De Vito & Gómez, 2020; Laing, 2020). It also led to a weak global economy worldwide (Goodell, 2020; Jin, 2021). In Malaysia, the government had imposed drastic lockdown measures with the first movement control order (MCO) on the 18 March 2020 when COVID-19 disease hit a drastic increasing trend of infected patients. The implementation of MCO was aimed at securing the economy and society through social distancing, self-isolation, and travel restrictions.

The sudden imposition of the MCO wreaked havoc on the food production and supply chains. The agricultural food supply chain mainly involves a few phases from the farm (producer) to the consumer. The restriction on movement, traffic, and market operation hours during the implementation of MCO had massively affected the food supply chains, especially for those in the urban areas (Surendan, 2020). Internationally, this had caused a shock due to the disruption in the supply (Peña-Lévano, Melo & Burney, 2020; Hayes et al., 2021) and shortage of workers (Villamarin Rodriguez & Pranay Kumar, 2021). In some other countries, despite having a sufficient food supply, farmers were hesitant to sell their produce, resulting in a quick rise in the prices of the products (Han, 2021). Meanwhile, the mass grain-production efficiency in Sichuan, Hunan and Hubei, China were restricted by manpower scarcity (Pu & Zhong, 2020). All COVID-19 affected countries were forced to take more drastic measures to prevent and curb the infection through the re-evaluation of their agri-food policies in order to secure the food availability and accessibility.

The establishment of the strong and dynamic agri-food supply chain with the involvement of cross-disciplinary experts and policy maker, is crucial to sustain the agricultural production and supply that had significantly impacted the livelihood of Malaysians. This review serves as the reference for policy maker, agri-food industry player, agronomist, information technology investor and cross-disciplinary researcher and expert.

## Materials and Methods

This study was carried out through a desk review of the secondary source of information covering the impact of COVID-19 in Malaysia particularly in the agri-food aspect, and a wide range of strategies and initiatives as the effective measures to overcome the crisis of this pandemic. Online desk research of the government published data and customer desk research were utilized to complete this study. Search engines such as Google Scholar and the statistical data from the official websites including the Department of Statistics Malaysia (DOSM) and the Food and Fertilizer Technology Center for the Asian and Pacific Region (FFTC-AP), were utilized. Keywords such as impact of COVID-19, pandemic, and agri-food supply were used to conduct the searches. The articles identified to be related to the study’s objective were then downloaded and included in the study. Descriptive methods were used as the primary analysis technique following the descriptive analysis and visual data analysis in performing the sources obtained.

## Scenario of covid-19 in malaysia

The SARS-CoV-2 spread rapidly from China to other countries, including Malaysia, before being classified as a pandemic on 12 March 2020 by the world health organization (WHO). As of early 2020, this outbreak started to spread faster worldwide than expected. The first confirmed COVID-19 positive case in Malaysia was reported on 25 January 2020, involving three Chinese citizens, and the first Malaysian COVID-19 patient was reported on 4 February 2020.

**Figure 1:**
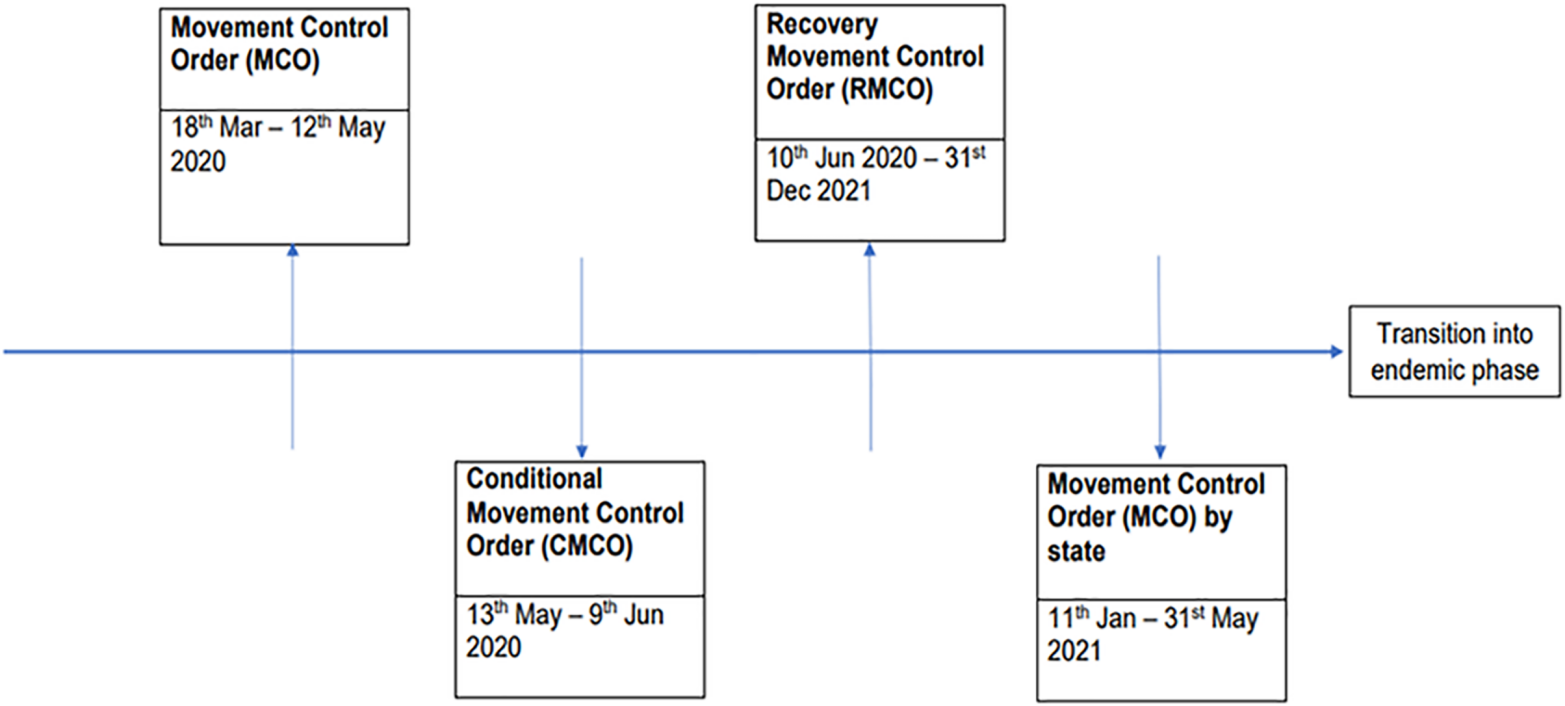
Timeline of MCO implementation in Malaysia during year 2020 to 2022. Started with the movement control order (MCO) to movement control order by state before entering into the endemic phase in June 2021.

Malaysia started imposing the movement control order (MCO) when the collective cases of COVID-19 had reached close to 800 on 18 March 2020. Throughout the year of 2020 to 2022, The government of Malaysia had established various movement control orders (MCO) to battle against the COVID-19 pandemic through extending the period of movement control order (MCO) or switching between the conditional movement control (CMCO), the recovery movement control order (RMCO) and the enhanced movement control order (EMCO) accordingly to the accumulated cases that were reported from time to time (Fig. 1).

During the first MCO, the public was thrown into chaos with the increase reported cases, and public awareness of the threat posed by COVID-19 exacerbated panic buying of foods and clinical equipment. For instance, dry or canned food supplies, instant noodles, cooking oil and mineral water were thought to be a rational choice of items hoarded during the lockdown (Yau et al., 2020). As a result, shortages of food with a long shelf- life were serious during this critical time (Begum et al., 2021). However, during these phases, even though new rules were introduced to prevent the spreading of the virus, agricultural activities in the Asia were greatly affected (Azra et al., 2021), including Malaysia. These measures were not economically friendly and severely disrupted the supply and demand chains in the agri-food industry.

Fortunately, most of the areas in Malaysia had entered into the Phase four of the national recovery plan (NRP) in the last quarter of 2021 with the total vaccination rate of adult citizens reaching 95.5%. This greatly stimulated Malaysia’s economy growth with a greater labour force by allowing the business and social activities to resume with extended duration of operation. The unemployment rate had declined from 4.8% in 2020 to 4.3% in 2021 (Department of Statistics Malaysia (DOSM), 2022a). Healthy employment growth continues to rise, and unemployment is expected to decline further.

In Malaysia, the primary food production industry was permitted to resume normal operations, including agriculture and fisheries. However, the disruption of transportation has significantly affected the agri-food chain. The government imposed various coping strategies to secure the food supply chain and curb the post-COVID-19 impacts. The Malaysian government introduced economic stimulus packages with a short-term recovery plan for relieving individuals’ and organizations’ burdens (Ministry of Finance of Malaysia, 2020). The financial support from the economic stimulus packages that targeted directly in agri-food production sectors is tabulated in Table 2.

**Table 2:** Financial support from the economic stimulus packages of the Malaysia government during the COVID-19 pandemic.

Government initiative	Total amount (RM)
Agriculture and food production grant for small and medium enterprises.	40 million
Food Security Fund for farmers and fishermen to increase domestic agri-food production.	1 billion
Development of infrastructure for food storage, distribution, and crop integration programs.	100 million
RM100,000 to RM200,000 for viable farmers’ associations and fishermen’s associations. This is to develop agro-food projects that are capable of generating income within 3 to 6 months.	–
Electricity bill discounts −15% discount on electricity bill for the tourism sector as well as a 2% discount for commercial, industrial, agricultural and household sectors in Peninsular Malaysia beginning 1 April 2020.	–

**Note:**

Source: Ministry of Finance of Malaysia, 2020; Prime Minister Office of Malaysia, 2020.

This pandemic had reflected the risk of agri-food security and threatened the social economy in most of the developing countries that are heavily supported by agricultural or related sectors. Therefore, it was crucial to keep the workers in the food supply chain protected and maintained especially during the COVID-19 crisis (Food and Agriculture Organization of the United Nations (FAO) & World Health Organization (WHO), 2020) and to ensure consumers’ assurance in food safety and security (Food and Agriculture Organization of the United Nations (FAO) & World Health Organization (WHO), 2020).

## Economic impacts on agricultural sector

### Agricultural food production

Agriculture sector is the main pillar in supporting the economy of Malaysia, with a contribution of 7.4% to the country’s gross domestic product (GDP) (Department of Statistics Malaysia (DOSM), 2022b). COVID-19 was reported to significantly affect agriculture in two major aspects: food supply and demand (Food and Agriculture Organization of the United Nations (FAO), 2020a). The economics of agriculture including crop farming, livestock, agroforestry, fishing and aquaculture, were not spared from the devastation of COVID-19. The restriction in movement during MCO had affected the hospitality sectors and the leisure industry, such as restaurants and commercial food services, thus resulted in the disruption of the global supply chain (Shaharudin, 2020). For instance, in Cameron Highlands, Pahang, Malaysia, the agriculture and tourism industries worth RM1.4 billion annually were greatly affected amid COVID-19 (Ishak, 2021), and vegetables were discarded due to logistic disruption.

During the pandemic, interruption in agri-food supply chain stability was critically caused by inconsistent supply of agri-food to the market due to restriction in movement. Another impact of COVID-19 due to the disruption of the food supply chain includes the delay in receiving inputs, reduction in cultivation, damage of fresh produce, lower productivity and decrease of farmers’ income. In 2020, Malaysia’s GDP fell by 5.6%, contrary to a 4.4% positive growth in 2019. The drop in the sector’s percentage caused by commodities such as palm oil, which was reported to have a negative growth of 3.6%. Nevertheless, it is still the most significant contributor to the agricultural sector, with values reported to be RM36.9 billion (37.1%). At the association of Southeast Asian nations ( ASEAN) level, the Malaysia agriculture sector’s contribution to GDP ranges 0.03–22.8%, with Cambodia and Myanmar each contributing 22.8% (Department of Statistics Malaysia (DOSM), 2021a).

**Figure 2:**
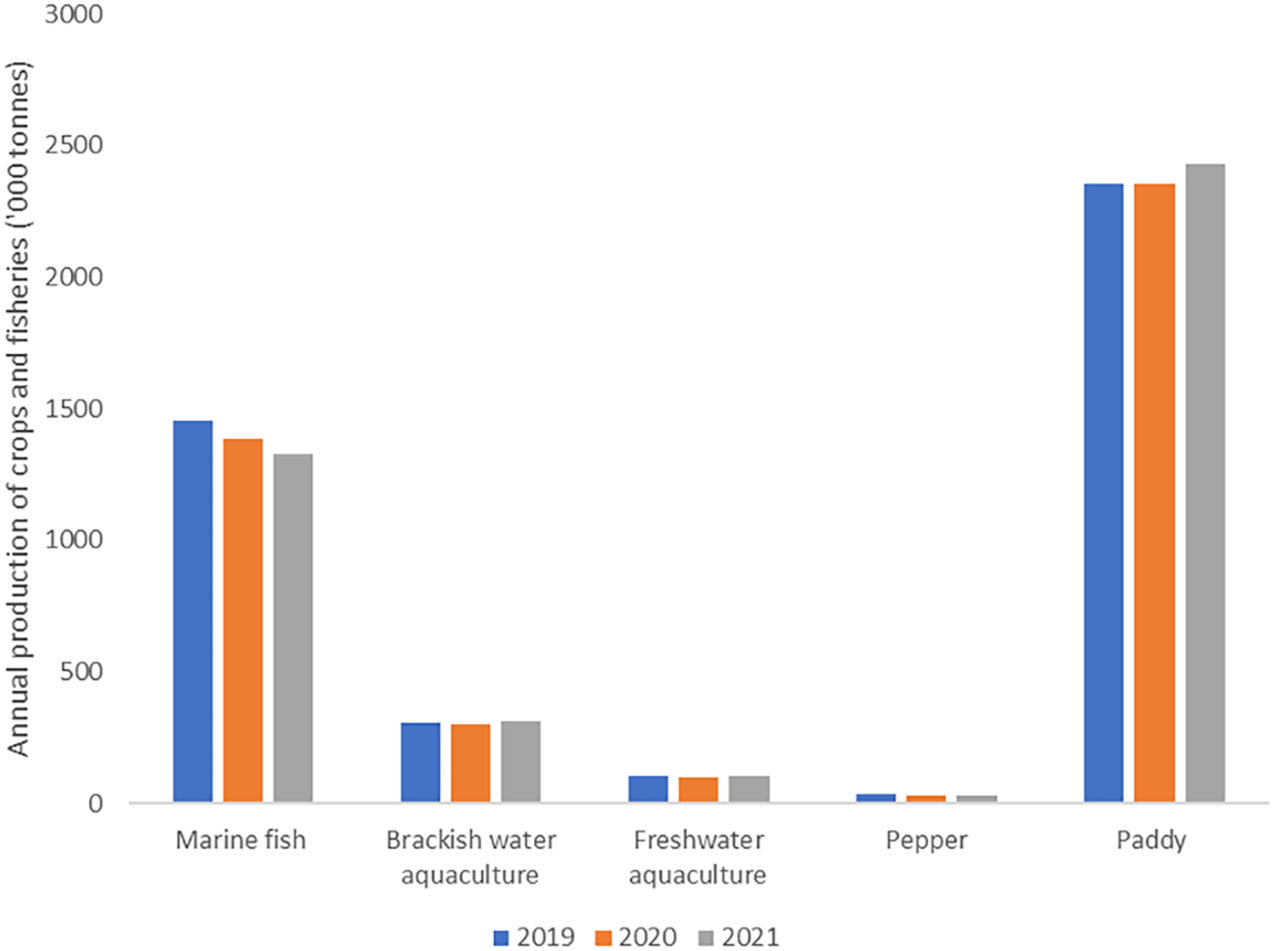
The impact of COVID-19 on the annual production of the selected crops and fisheries in Malaysia between 2019 and 2021. The figure was created by the authors from the data of the Department of Statistics Malaysia (DOSM) (2021d).

Agriculture products such as paddy, natural rubber, freshwater aquaculture, brackish water aquaculture and marine fish had decreased from the year 2019 to 2020 (Department of Statistics Malaysia (DOSM), 2021a) (Fig. 2). The annual productivity had slightly increased in the following year (2021) except for marine fish. Generally, Malaysia’s producer price index (PPI) declined from 2019 to 2020. However, in October 2020, there was an increase in the PPI of local production. This was a subsequent in the increase of the mining index with 71.2%. The increase in the price of crude oil (79.8%) and natural gas (38.2%) also led to the rise of PPI index. The agriculture, forestry, and fishing index also experienced an increase with only 19.1%. The increase in the production of palm oil, poultry and poultry’s produce (egg) contributes to the rise in the agricultural index (Department of Statistics Malaysia (DOSM), 2021b).

Generally, the production of commodity crop was reported lower in 2020 than 2019. Malaysia is known to be the second largest palm oil producer and exporter globally, accounting for 26% of the world production and 34% of the world exports in 2020 (International Trade Administration (ITA), 2021). In 2021, the production of crude palm oil had declined by 1.02 million tonnes (Malaysian Palm Oil Board (MPOB), 2022a), however it was still the highest compared to other agri-commodities’ production. The total production of palm oil in the year 2021 was lower than 2020 but higher in price (Malaysian Palm Oil Board (MPOB), 2022b). The reduction in palm oil production was associated to the labour shortage that had caused RM6 to RM9 billion loss in 2020 and 2021, respectively (Izmir, 2021). This scenario was mainly due to the return of foreign workers to their home country resulting from the closing of the border by the Malaysian authority and some were caused by invalid work permits (Mei, 2021). To cope with this problem, the government of Malaysia had decided to absorb 32,000 fully vaccinated foreign workers into the plantation sectors (Izmir, 2021).

Labour shortage following by the restriction in mobility during the COVID-19 pandemic had resulted in the decline of another agri-food production in Malaysia. In 2020, the production of pepper was reported to decline 3.1 thousand tonnes and fruit production fell by 0.7% over the same period. On the other hand, the operation of agri-production system that involved local labour was not significantly affected. For instance, Malaysia’s vegetable production increased by 0.9%, with Pahang being the top producer with 35.1%, followed by Johor (20.2%) and Kelantan (12.5%). Johor was recorded as the most fruit-producing state in Malaysia contributed 36.0%, followed by Pahang (13.1%) and Sarawak (12.0%) (Department of Statistics Malaysia (DOSM), 2021a).

The world is moving backwards in the efforts to end hunger, and the impact of the COVID-19 pandemic has further deteriorated the food security and nutritional status of the vulnerable population (Food and Agriculture Organization of the United Nations (FAO), 2020b). In Malaysia, rice and cattle were mainly for the domestic consumption, while commodity crops were mainly for exports. Palm oil, cocoa, pineapple, and pepper are among the main export crops covering roughly 75% of cultivated land. The government meanwhile encouraged farmers to target on the higher-value crops. This strategy threatened the national sustainable rice production system in maintaining the domestic rice self-sufficiency level at 65–70% (Mohamed Arshad et al., 2011).

The crisis of post-COVID-19 had shown several unsustainable aspects of agricultural production and supply chain models. Agriculture production is almost entirely dependent on migrant workers and relies on imported fertilizers and animal feeds. In 2020, RM4.6 billion was spent for animal feed and RM3.2 billion for fertilizer (Department of Statistics Malaysia (DOSM), 2021c). The travel bans and the border closure were greatly bound to cause problems such as labour shortages, disruption to food supplies, food hoarding, logistics and transportation and hence, resulted in the decrease in trading efficiency (Organisation for Economic Co-operation and Development (OECD), 2020). Therefore, the government of Malaysia was urged to embrace comprehensive planning to transform the current agriculture model into a more dynamic, flexible and sustainable in the near future. The review of the trade policies and cooperation at the international level are important to ensure agri-food availability in stabilizing the food supply chain.

### Agricultural food supply chain in Malaysia

**Figure 3:**
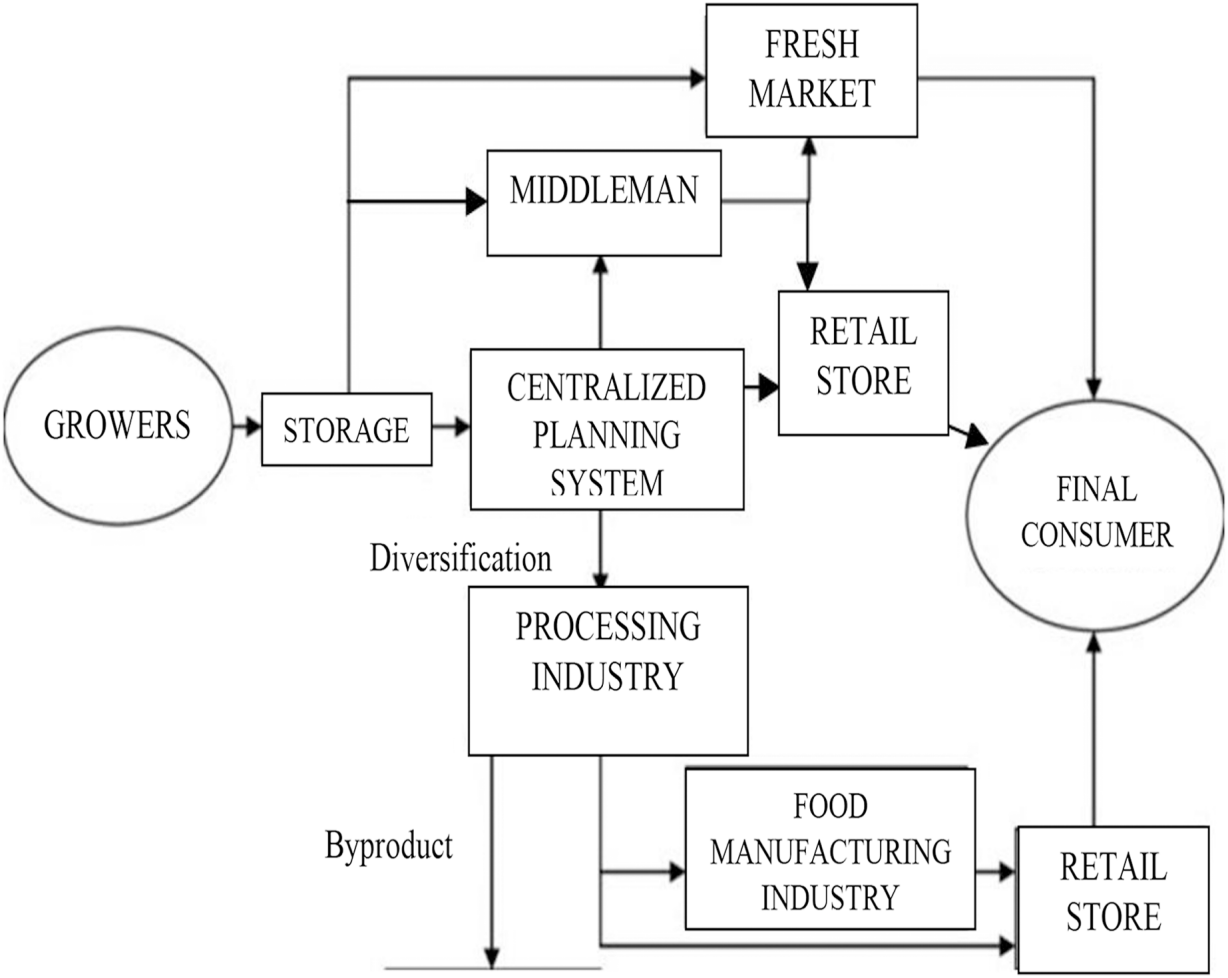
Flow diagram of the agricultural food supply chain in Malaysia that involved huge range of activities from grower, wholesaler and manufacturer before reaching to the final consumer. Modified from Yok et al. (2016). (CC BY-NC-ND 4.0) https://creativecommons.org/licenses/by-nc-nd/4.0/.

The agri-food supply chain holds a momentous role in driving the economy of Malaysia, which contributes to an important proportion in the national GDP. In Malaysia, the agri-food marketing system involves a dynamic and huge range of activities in the supply chain procedures, as summarized in Fig. 3 (Yok et al., 2016). Generally, this conventional supply chain was considered loose, fragmented, duplicable in function and unstable (Arshad, Mohamed & Latiff, 2006) even before the hit of COVID-19 pandemic. The application of a centralized manufacturing system in the food chain was risky and caused significant disruption during the COVID-19 pandemic. In fact, centralization aimed at increasing production and reducing costs. However, it triggered rigidity and prolonged food supply chain issues (Aday & Aday, 2020). Additionally, using a small number of large manufacturer facilities to achieve food demands might jeopardise the supply chain (Almena et al., 2019). This was evident especially during MCO implementation, which led to the closure and break of the production line.

Throughout the COVID-19 outbreak, many countries imposed total lockdown and movement restrictions to curb the infection. The quarantine policies for importing and exporting agri-food within countries had worsened the impact of the pandemic on the agri-food supply chain. During the pandemic of COVID-19, the agri-food supply chain involving two major aspects: food supply and food demand, had considerably influenced and impacted food security. The perspectives of food security during the COVID-19 crisis included availability (supply), accessibility, utilization, stability (Zurayk, 2020) and food safety (Food and Agriculture Organization of the United Nations (FAO) & World Health Organization (WHO), 2020). The COVID-19 crisis had affected food security and supply chain in both supply (production capacity and distribution) and demand (consumer behavior) aspects. Disruptions in the complex web of the food chain created simultaneous surpluses for producers and shortages for consumers.

**Figure 4:**
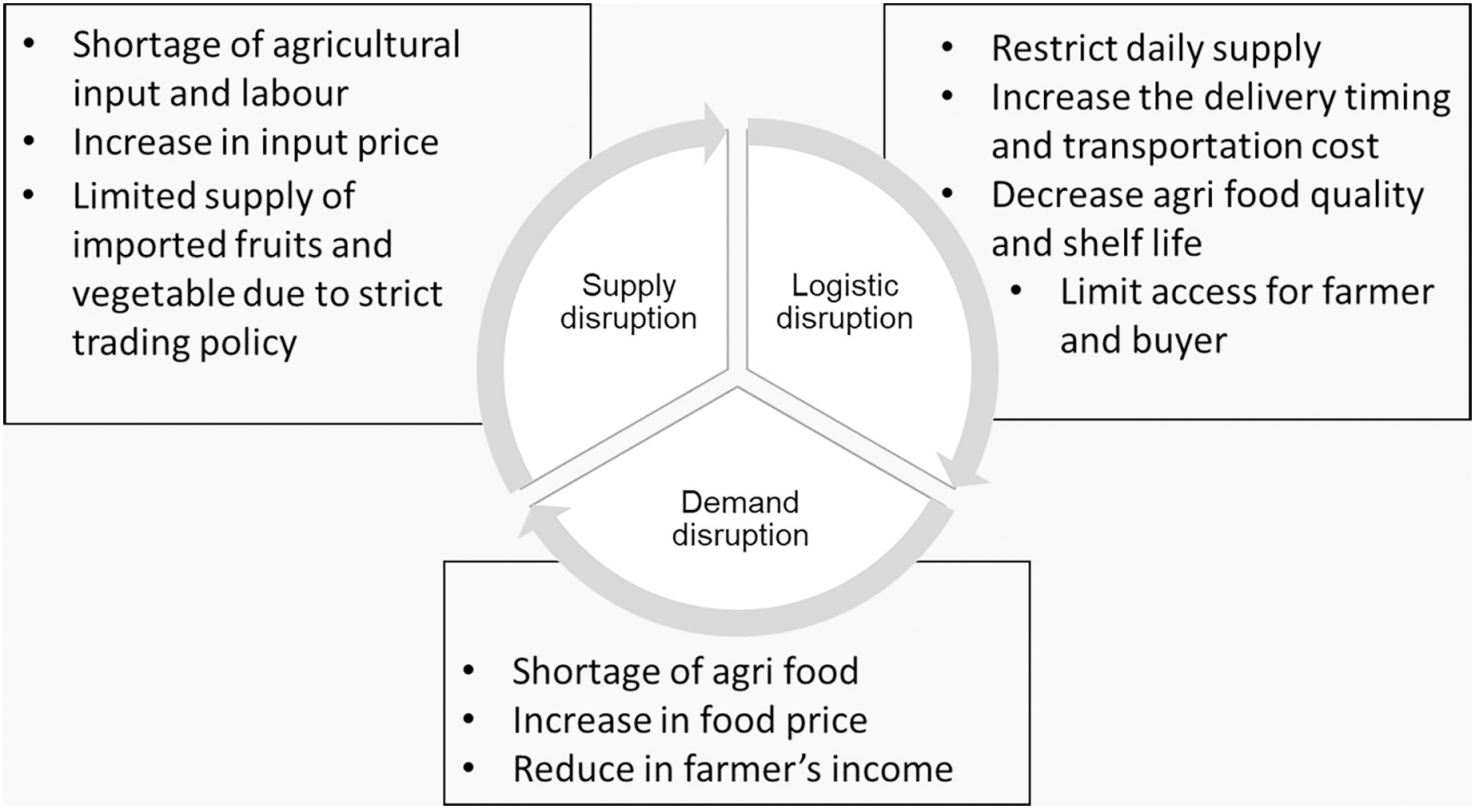
The impact of COVID-19 on the agri-food supply chain due to disruption from the supply, logistic and demand sides, resulted food shortage and food price spikes. Data modified from Mishra, Singh & Subramanian (2021).

Food availability and accessibility became more vulnerable during the COVID-19 pandemic. The enforcement of MCO nonetheless should not affect the food chain activity. Nevertheless, the agri-food supply chain and the marketing system were undergoing a drastic transformation forced by the implementation of MCO. The challenges in handling the demand of consumers, overcoming food production, issues due to lockdown, minimum workers, limited operation hours, and strict import and export trade policies were critical (Alsuwailem et al., 2022). The restriction of movement disrupted the supply, logistics and demand sides and triggered various challenges in the food supply chain (Fig. 4). The food supply chain in Malaysia is highly dependent on labour for its operations, from the production to the marketing. Labour shortage during COVID-19 crisis disrupted the dynamic network in food supply chain and limited food accessibility. In order to manage these issues, the government of Malaysia should develop and embrace an effective food supply chain to meet the demands of consumers and secure food safety at the same time.

### Labour force

Agriculture sectors employed approximately 10.28% of the total employment in Malaysia in 2019 and contributed to about 8% of the country’s GDP (The World Bank, 2022). During the outbreak of COVID-19, the high rate of transmission among workers’ health and labour shortage had been the major issues in the agricultural industry. Workers from low- and middle-income countries with little to no savings were obliged to work despite the self-isolation protocol during the COVID-19 pandemic. Hence, these workers were at high risk of COVID-19 infection (Occupational Safety and Health Administration (OSHA), 2020). In Malaysia, a standard operation procedure (SOP) had been established to protect the workers in the palm oil and rubber sectors towards the fulfillment of consumers’ demand (Abdul Aziz, Mohd Sukor & Ab Razak, 2020).

Besides, the travel ban had contributed to a shortage of seasonal and temporary farm workers (International Organization for Migration (IOM), 2020) and led to a workforce shortage in most farm producers. There was a rapid increase in employment losses around the world. International labour organization (ILO) estimated that the COVID-19 pandemic had affected 81% (2.7 billion workers) global workforce due to full or partial closure of the workplace (International Labor Organization (ILO), 2020). In order to sustain the food demand, agriculture was one of the sectors that was required to be kept functional. However, the mobility restrictions had kept seasonal and temporary workers from travelling to their workplace and hindering the productivity of this essential sector (Kalantaryan, Mazza & Scipioni, 2020).

**Figure 5:**
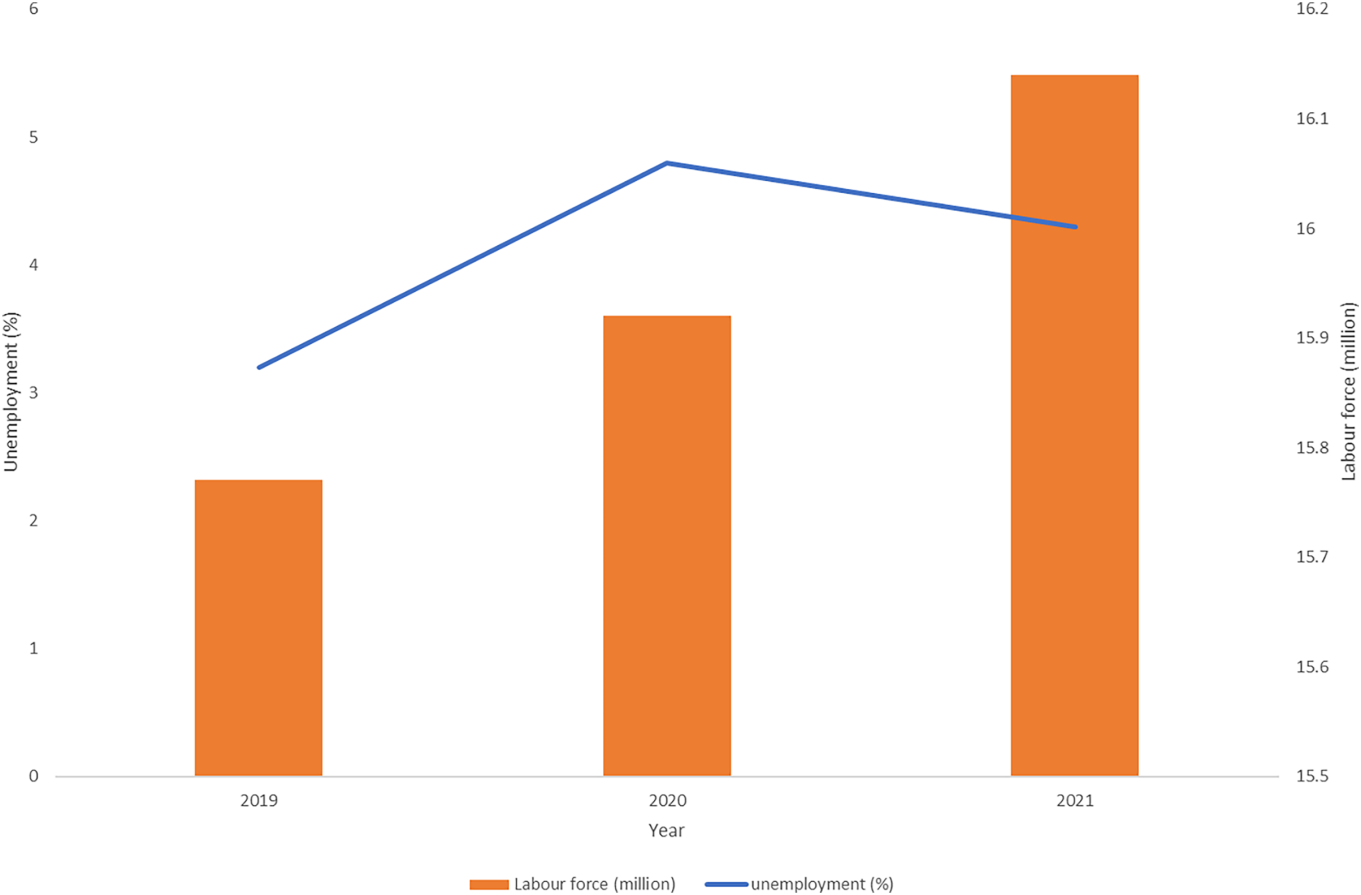
The unemployment percentage and labor force in Malaysia during COVID-19 crisis, 2019 to 2021. The unemployment percentage was the highest (4.8%) in 2020 with labor force achieved 16.1 million in 2021.

In Malaysia, it was reported that unemployment reached 4.8% in the last quarter of the year 2020 compared with 3.2% in 2019 and 4.3% in 2021. The labour force of Malaysia, however, is on the increasing trend from 15.77 million in 2019 to 15.92 million in 2020 and 16.14 million in 2021 (Department of Statistics Malaysia (DOSM), 2022a) (Fig. 5). Interestingly, the annual employed percentage in the agricultural sectors increased from 1,541.1 thousand people in 2019 to 1,566 thousand in 2020 (Department of Statistics Malaysia (DOSM), 2021a). This was mainly due to the restriction in the operation of manufacturing, tourism, and hospitality industries during the implementation of MCO, which impacted the diversion of the labour force into the agriculture sector.

**Figure 6:**
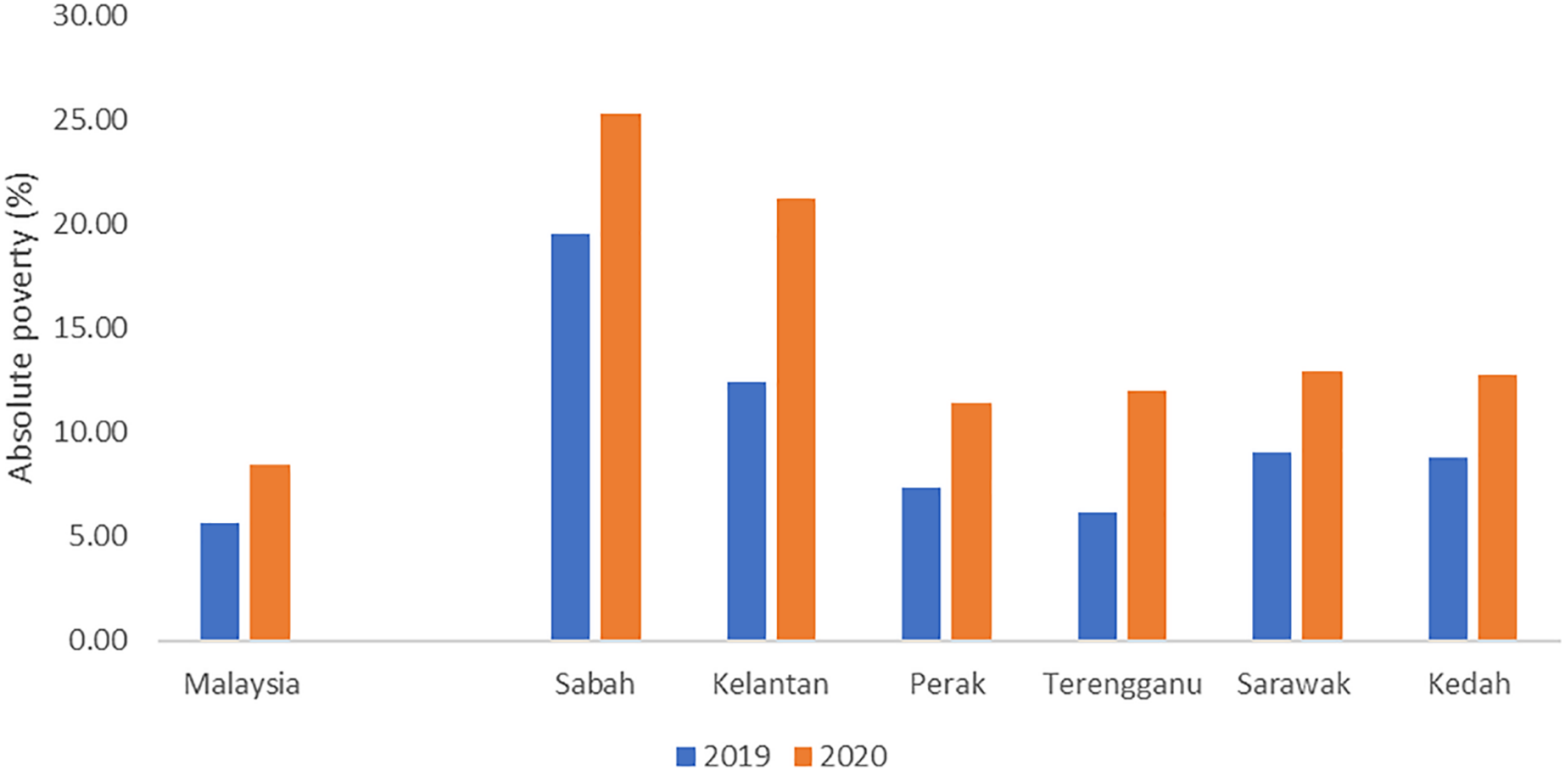
The incidence of absolute poverty in Malaysia and by selected state in 2019 and 2020. The increase of incidence in the national absolute poverty caused by the consequences of the COVID-19 pandemic.

This crisis seriously affected the overall Malaysia’s economic activities, which can be directly reflected in the country’s absolute poverty (Fig. 6). The absolute poverty in Malaysia increased from 5.6% in 2019 to 8.4% in 2020, significantly due to the increasing rate of unemployment (Department of Statistics Malaysia (DOSM), 2022a). Moreover, paid employment and self-employment were reported to decrease by 16.1% and 9.7%, respectively, in 2020. The mean monthly household gross income had reduced by 10.3% to RM7,089 in 2020 compared to RM7,901 in 2019. In terms of median, the monthly household gross income declined 11.3% in 2020 compared to 2019. There was an additional 12.5% of households with income of less than RM2,500.

The recent COVID-19 pandemic has again highlighted the importance of food security in Malaysia. During the outbreak of COVID-19 in the year 2020, only 19 items of agricultural commodities were recorded for more than 100% self-sufficiency ratio (SSR) compared to 25 items in the year 2019. The declination of 24 out of the 45 agri-food in import dependency ratio (IDR) was contributed by the external trade restriction crisis. The reduction in production was the implication of the restriction on the operation hour with a minimum number of employees (Department of Statistics Malaysia (DOSM), 2021c). Hence, the Ministry of Plantation Industries and Commodities, together with the Department of Agriculture of Malaysia, were urged to take appropriate measures to cushion the impact of the COVID-19 crisis towards reviving household income by stabilizing the agricultural-related sectors by providing more employment opportunities. Strategies and policies were needed to be established and revised, aiming to strengthen the agricultural production system and stabilizing the food supply chain.

## Post-covid-19 opportunity for reflection

**Figure 7:**
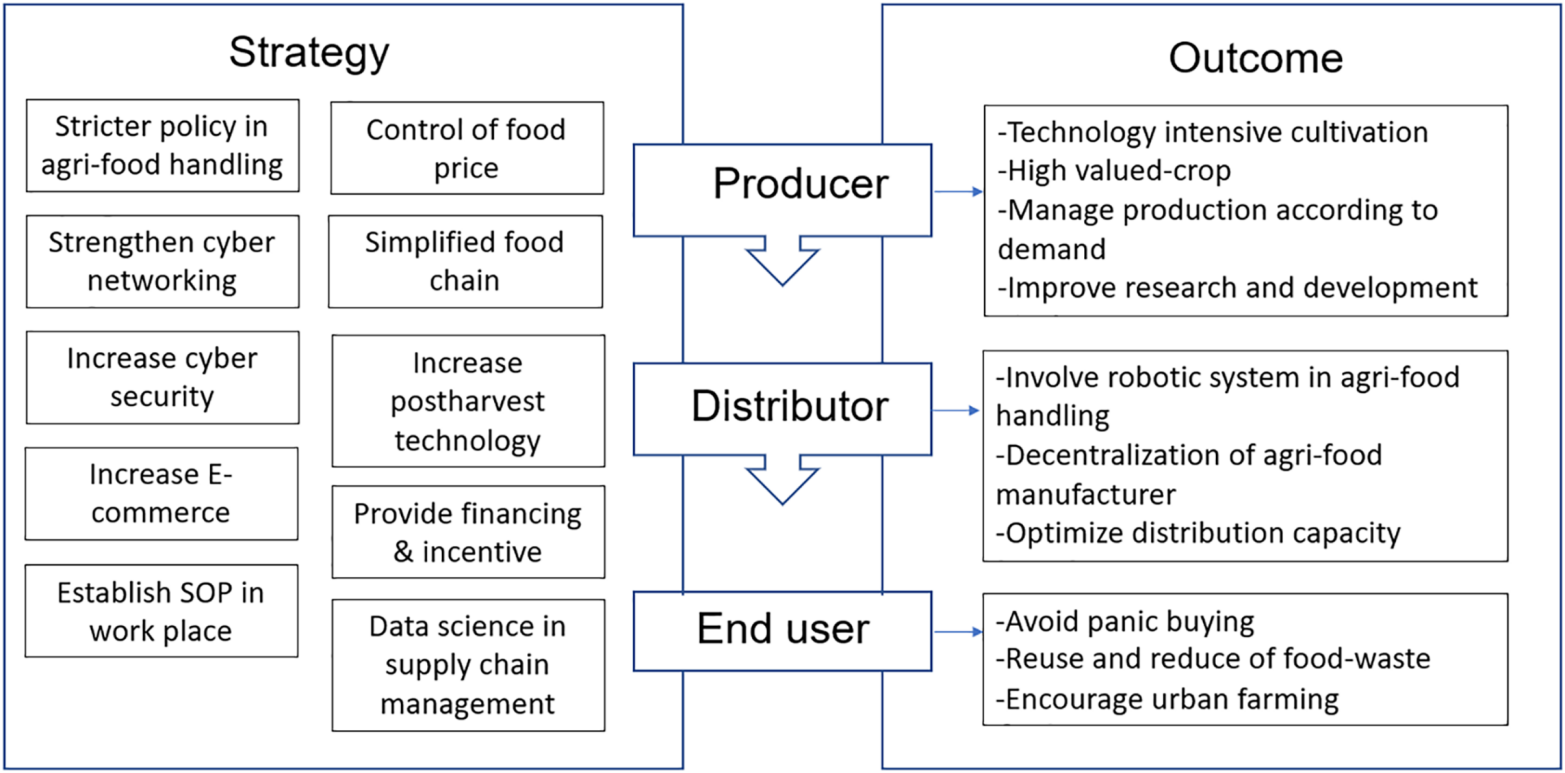
Flow diagram of the potential strategies implemented to the key players and the outcomes obtained from the food supply chain in Malaysia to secure from the crisis of COVID-19 pandemic.

The pandemic had reflected the instability of the agri-food supply chain in Malaysia. During the pandemic of COVID-19, the disruption in logistics and transportation substantially impacted the accessibility of consumers to agri-food supply, created a panic purchasing trend and led to the spike in agri-food prices. An increase in agricultural imports in Malaysia is indeed not a sustainable measure to combat the potential upcoming health crisis. However, the crisis of COVID-19 created an opportunity for developing better flexible approaches in handling emergencies on agri-food production and supply chains. Therefore, a comprehensive measure involving the policymakers, growers, food manufacturers, wholesalers, research institutions, private and government sectors, retailers, transporters, engineers, and consumers need to be integrated to establish and strengthen the agri-food supply chain (Fig. 7).

### Government preparation to rejuvenate the agriculture sectors

The government’s role in revitalizing the agri-food sectors in Malaysia to cushion the impacts of this crisis is important through the strengthening of various development plants. The implementation of the national food securities framework and national agro-food policy 2021–2030 along with the strategies and initiatives, help to achieve the sufficiency of food supply and compliance with international food safety standards (Dardak, 2022). The financial plan under Malaysia Budget 2021 aimed to revive the economy and helped Malaysians, especially those who were affected by the COVID-19 pandemic (Malaysia Government, 2021). Under Budget 2021, the government put more efforts on encouraging the growth of the economy.

The Malaysian government has also identified new directions and strategies that can spearhead the development of agriculture under the 12^th^ Malaysia Development Plan. Under this plan, the agriculture sector is projected to grow approximately 3.8% annually and contributed about 7.0% to the GDP with emphasis on the transformation into a dynamic and competitive sector supported by research, development, commercialization and innovation (Dardak, 2022). Based on this direction and together with the industrial revolution 4.0, which focused on digital technology, the government should encourage and convince the producers to accept and adopt the digital platform to drive their business in exploring new markets, enhancing competitiveness and reducing dependency on the middleman (Amir et al., 2020).

**Figure 8:**
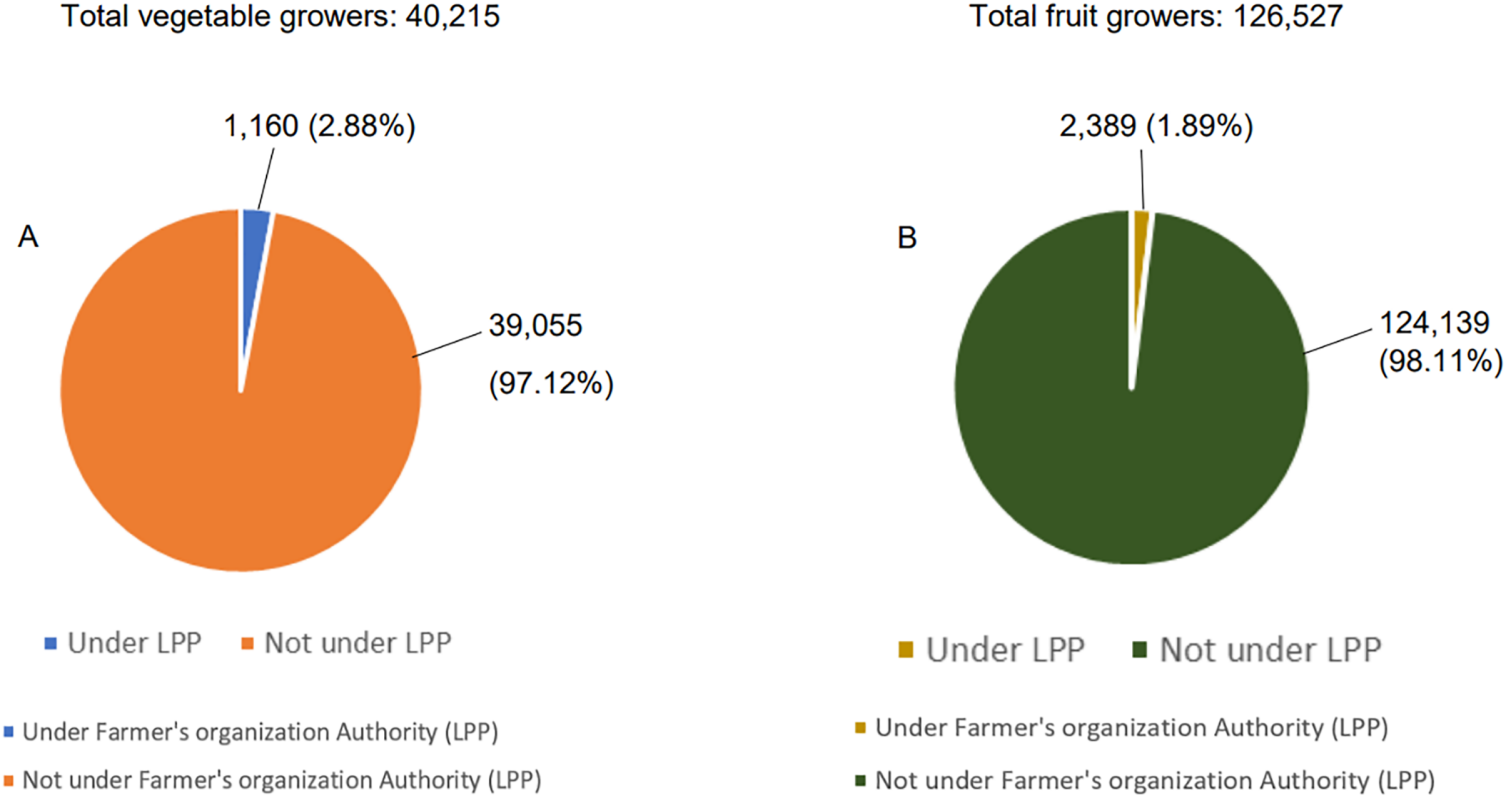
The number of vegetables (A) and fruits (B) growers in Malaysia (excluding Sarawak) under the farmer’s organization authority is relatively low in 2017.

The financial allocation for food security fund during the COVID-19 outbreak should be prolonged and continued to boost domestic agricultural and fishery production through various forms of assistance. Furthermore, the coverage of fund for the recipients should not only be limited to those registered under the area farmer’s associations (PPKs) and the area fishermen’s association (PNKs). This was because the funds were only allocated for farmers and fishermen under the PPK and PNK, whilst those beyond the associations were neglected (Shaharudin, 2020). According to the data obtained from the Lembaga Pertubuhan Peladang (LPP) (2017), only less than 5% of the total vegetable and fruit farmers were under the farmer’s organization authority (LPP) (Fig. 8). In order to achieve the objectives, the authorities should closely monitor and strengthen the agri-food production line to meet the demand and to ensure the supply chain is uninterrupted.

Besides, the government plays catalytic role in encouraging new energy into the agriculture business. The effort of ministry of agriculture and food industry Malaysia, through the national entrepreneurial group economic fund, was effective to rejuvenate the national agriculture sector through implementation of various measures to attract the involvement of young *agropreneur* (Mat Nawi, 2021). This program had funded 750 young *agropreneurs* in 2021 with RM15 millions allocation. The government was urged to establish a special committee, such as the immediate action unit, that can respond to and deal with the market chain issues, especially in relation to agriculture sectors (Amir et al., 2020).

### Integrated advanced technology in the food supply chain

Adopting technology in agri-food production systems through the application of technology-intensive systems and environmentally friendly innovations, such as the internet of things (IoT) concept, is the key to meeting the high productivity and sustainability goals in rejuvenating economic growth. IoT was introduced during the Industrial Revolution to ease data collection and transfer over a network using the internet (Ernita, 2015). The use of IoT in agriculture has recently diversified into the smart farming system, logistic and supply chain management towards visibility (Abideen et al., 2021), transparency, efficiency, accuracy, cost-effectiveness (Legchekov, 2022) and reduced dependency on labour. The role of IoT in strengthening the supply chain is by empowering reliable, consistent, up-to-date data to all the stakeholders (Zhang, Zhao & Qian, 2017) with the concept of visibility from the producer to the consumer in the supply chain (Somapa, Cools & Dullaert, 2018).

E-commerce has become an important platform accelerating digital transformation since the COVID-19 pandemic. In Malaysia, the government has established the national E-commerce Council to drive the implementation of the national E-commerce Strategic Roadmap 2.0’s. Furthermore, the government should proactively and consistently promote the accelerated adaptation of e-commerce through campaigns, training, and realignment of the existing incentive for micro, small and medium enterprises. The government’s initiative to strengthen and facilitate cross-border E-commerce aimed at widening the global market is the key to accelerating the digitization of the dynamic supply chain in Malaysia. Nevertheless, the rapid growing trend of e-commerce in Malaysia has caused significant challenges due to the rapid transformation from a physical to a digitized supply chain. Therefore, the adoption of IoT in the agri-food supply chain is necessary.

Efforts to rejuvenate the food-based agriculture sectors through IoT technology and intelligent big data analytics in the management of the supply chain allow predictable markets, improved operational marketing efficiency and enable timely decisions making to avoid unnecessary losses (Tan et al., 2021). Sharing the big data of the supply chain management within the key players in the supply chain leads to precision in delivering agri-food from producers to consumers.

**Figure 9:**
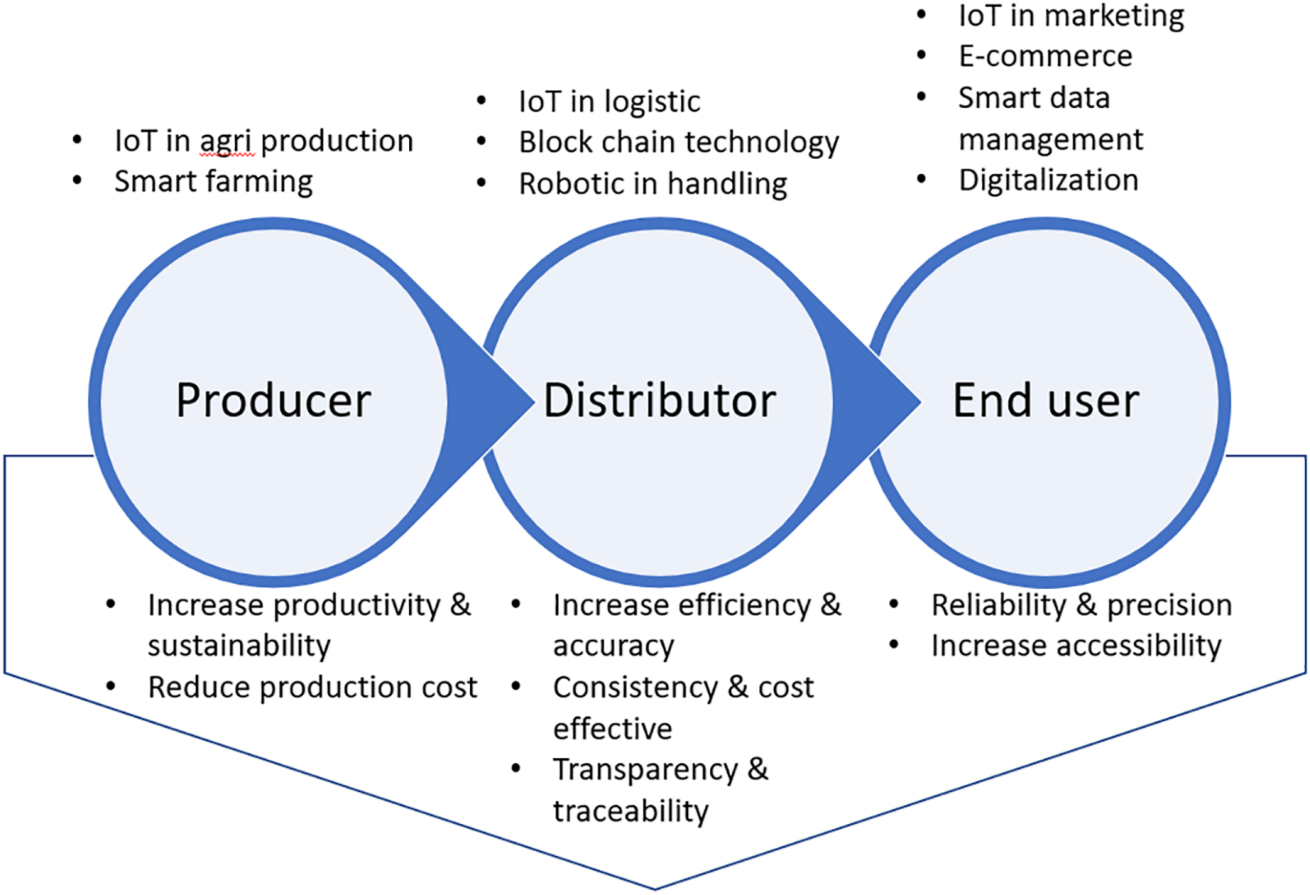
Integrated advanced-technology using internets of things (IoT), digital networks, robotics and intelligent data analytics to enhance the efficiency and flexibility of agri-food supply chain to invigorate post-COVID-19 economic growth.

Undeniably, the increasing manufacturing of Malaysian agricultural resources has become considerably sophisticated, and food safety is a great concern for the consumers, especially after the COVID-19 crisis. The issues of information transparency and ensuring the trust between producers and consumers in the supply chain are relatively important in the food supply chain. The implementation of blockchain technology enables visibility in supply chain management (Rogerson & Parry, 2020), where a ‘block’ is an information package that contains all the preliminary information and the new data (Lyasnikov et al., 2020). This technology can help to overcome the problem of transparency, security in stock management (Kshetri, 2018), improve the reliability of operation, secure the confidential transaction and availability of information, and reduce the interaction timing between participants in the supply chain (Lyasnikov et al., 2020). Blockchain technology reduces and minimizes the material flow parameter due to the change in management paradigm, where the decision is decentralized (Tapscott & Tapscott, 2017). Integration of technology-oriented, digital networks, robotics and intelligent data analytics with physical processes are crucial to enhance the supply chain efficiency and invigorate post-COVID-19 economic growth (Fig. 9).

### Research and development

Intensive progress in research and development involving the partnerships between private and government institutions is crucial in solving the problem experienced during the COVID-19 pandemic. To sustain the food security issues in the post-COVID-19 era, besides the increase in agri-food production, the recycling and regeneration of food waste to be reutilized in the food chain possess huge potential. According to Aldaco et al. (2020), food waste generation at the household level increased by 12% during the COVID-19 pandemic. The valuable bioactive compounds: pectin, flavonoids, and essential soil derived from food waste are possible to be reutilized as a preservative, gelling agent and food supplement (Galanakis, 2013). The research and development on the innovative extraction, fractionation and stabilization of these potential compounds toward cost and labour effective in the food production system are much encouraged.

Research collaboration with private sectors in reducing the dependency on the government effectively boosts the high productivity and efficiency in the agri-food production system through technology-intensive and environmentally friendly innovation approaches. For instance, the application of robotics technology enhances food safety in the food processing, manufacturing, servicing, and packaging lines. Robotic machines can lower the risk of human disease infection, reduce labour dependency, and solve human resource management issue (Carnevale & Hatak, 2020).

In addition, the role of government institutions such as the Department of Agriculture Malaysia, and the Malaysian Agricultural Research and Development Institute (MARDI) should highlight the plantation cultivation system of agri-food to replace the conventional and small scaled production system. The surge in consumer demand of high-quality agri-food has offered great opportunities for post-harvest technology development and quality management of agri-products in the food supply chain. The dissemination of the research outcomes, and relevant and appropriate knowledge to the targeted stakeholders in the food supply chain aid in accelerating the transformation towards meeting high productivity and sustainability.

## Conclusions

The pandemic of COVID-19 had caused global food security and safety concerns. During the pandemics, the network in agri-food chain was noticeably interrupted. The restrictions that the governments had imposed on the mobility of labour, tightening the import and export policies had impacted the drastic declination in agricultural production. The pandemic has reflected and reminded us that a stable and flexible agri-food supply chain is crucial to sustain food security. Better strategies and policies are needed to be established in strengthening the agricultural production system. Research and development in agricultural technology, such as IoT, mechanization, big data management help to reduce the dependency on foreign labour and enhance efficiency in agri-food supply chain. Technology in optimization used for agricultural land *via* crop and livestock integration needs to be further emphasized to increase the efficiency of land usage. Risk management analysis should also be conducted to reduce the uncertainty and enhance sustainability in the agri-food supply chain. Collaboration within countries, industries and individuals in various mechanisms helps to strengthen the stability of agri-food supply chain globally. Therefore, the government should be ready with the roadmap and enforce the measures to control the pandemic without disrupting the agri-food supply chain in the near future.

## Additional Information and Declarations

### Data Availability

The following information was supplied regarding data availability: This is a literature review.
